# Editorial: Nanotechnology for natural products

**DOI:** 10.3389/fchem.2022.1069892

**Published:** 2022-11-02

**Authors:** Jingshan S. Du, Tongkai Chen, Fang Liu, Shengpeng Wang, Jinming Zhang, Ruoyu Zhang

**Affiliations:** ^1^ Physical Sciences Division, Pacific Northwest National Laboratory, Richland, WA, United States; ^2^ Science and Technology Innovation Center, Guangzhou University of Chinese Medicine, Guangzhou, China; ^3^ School of Pharmaceutical Sciences, Guangzhou University of Chinese Medicine, Guangzhou, China; ^4^ State Key Laboratory of Quality Research in Chinese Medicine, Institute of Chinese Medical Sciences, University of Macau, Macao SAR, China; ^5^ State Key Laboratory of Southwestern Chinese Medicine Resources, School of Pharmacy, Chengdu University of Traditional Chinese Medicine, Chengdu, China; ^6^ Institute of Engineering Medicine, School of Medical Technology, Beijing Institute of Technology, Beijing, China

**Keywords:** nanotechnology, natural products, drug delivery, conjugates, liposomes, aptamers, peptides, nanoparticles

Humans around the globe have lived through an unprecedented time. The emergence and rapid spread of coronavirus disease 2019 (COVID-19) have resulted in the loss of millions of lives, and this number is still climbing every day and hour. As a consequence, there have been drastic changes over the past 2 years in how we live, communicate, and work; most importantly, COVID-19 has raised global awareness of infectious diseases and pandemic responses to an exceptional level. Like COVID-19, highly contagious and lethal viruses, such as the human immunodeficiency virus (HIV), Ebola, and the recent addition to the list, Monkeypox, pose extraordinary challenges to modern civilizations. On the other hand, non-communicable diseases, including heart disease, stroke, and cancer, are still claiming the majority of deaths on a global level (World Health Organization, 2020).

Before the availability of synthetic drugs, civilizations worldwide relied on natural products extracted from the environment and various organisms for healthcare. Natural products and their derivatives continue to be critical components in the drug library available today. Indeed, almost a quarter of the new drugs approved by the United States Food and Drug Administration (FDA) since 1981 are natural products, botanical mixtures, or their derivatives (Newman and Cragg, 2020). Recent developments in analytical characterization, genome mining, and engineering have further increased interest in drug discovery from the pool of natural products (Atanasov et al., 2021).

This Research Topic celebrates a collection of studies at the intersection of natural product research and nanotechnological advances. As a successor and extension of the Research Topic in “*Nanotechnology in Traditional Medicines and Natural Products*” published last year (Zhang et al., 2021), the Research Topic focused more on recent developments in employing the synthesis and characterization of nanostructures for the delivery of functional natural products. Specifically, the use of carrier nanostructures to conjugate or encapsulate therapeutically active natural products with carrier nanostructures could enable their robust, targeted delivery tailored to either tackle a specific disease or unlock a drug administration pathway. Over the last decade, nanostructures such as liposomes (Pattni et al., 2015) and lipid nanoparticles (Hou et al., 2021) have been developed as versatile platforms for the inclusion and delivery of a wide range of therapeutical compounds; a significant advance in this aspect is the stabilization of mRNA strands for COVID-19 vaccination recently produced by Pfizer/BioNTech and Moderna (Tenchov et al., 2021). Such engineered nanomaterials are now widely used in the development of natural product-based and synthetic drugs, and recent studies have primarily focused on increasing the bioavailability of natural products, enhancing the targeting efficacy, and enabling the controlled release of active components by nanocarriers (Watkins et al., 2015). However, regulatory forces such as the United States FDA remain cautious with the evaluation of each product on a case-by-case basis (Wang and Grainger, 2022) due to the often under-addressed issues with potential toxicity and stability *in vivo*.

In this Research Topic, Zeng et al. provided a timely review of natural products conjugated to and contained within nanomaterials for the ongoing COVID-19 pandemic. Natural products that are potentially useful in the mitigation of this disease, such as chloroquine and curcumin, supported in nanocarrier systems, have been discussed in detail. More broadly, Cheng et al. comprehensively reviewed the use of liposomes as a nanocarrier for natural products. This article provides a summary of natural product compounds and extracts used to form liposomal structures. Readers can further enjoy the practical aspects of these engineered drugs for the treatment of specific diseases as well as insights into future directions in this field.

In addition to inclusion in liposomal compartments, conjugation with proteins/peptides and nucleic acids also effectively enables targeted and sustained delivery of natural products. In a mini review, Chen et al. summarized recent advances in the chemistry and application of DNA-conjugated natural products for therapeutics. This article begins with the synthetic strategies to conjugate several types of natural products to nucleic acids through either terminal, backbone, or nucleobase site modification. The therapeutic use of such aptamer structures was discussed, and important insights into their challenges and opportunities were provided.

Three articles in this Research Topic collection specifically demonstrated how the development of nanocarrier systems helps to address challenges in the delivery of natural products through different pathways. In their original research article, Xiao et al. implemented an edible delivery system by loading isoliquiritigenin (ISL) on zein/caseinate complex nanoparticles. These structures increase the stability of ISL and prevent its early release in the stomach, which facilitates the efficacy of colonic inflammation treatment in mouse models *in vivo*. Razavi et al. provided a comprehensive review of recent efforts to construct nanodelivery systems to overcome ocular barriers and enable effective ocular delivery of therapeutically active natural products. In this article, the authors discussed barriers in corneal and non-corneal delivery pathways. Recent studies using polymer nanoparticles, micelles, nanofibers, dendrimers, lipid nanoparticles, liposomes, and niosomes to overcome these barriers and improve the effectiveness of drug delivery to ocular target sites were reviewed. The original research article by Dang et al. reported the modification of liposomes with a polyethylene glycol-conjugated nuclear-targeted peptide to enable nuclear-targeted delivery of gambogic acid. These engineered liposomes showed evidenced targeting and treatment of tumor tissues in a mouse breast cancer model.

The chemistry involved in the synthesis, functionalization, and modification continues to be a central topic in the development of new nanoengineered drugs. We hope that this Research Topic will serve not only as a collection of studies in the past but also as a forward-looking guide to what is possible in the cross-disciplinary field of nanotechnology-enabled natural product research.
